# Burden and predictors of advanced HIV disease among people living with HIV at Zewditu Memorial Hospital in Addis Ababa, Ethiopia

**DOI:** 10.3389/fpubh.2026.1837932

**Published:** 2026-06-10

**Authors:** Million Hailu Tesema, Azwihangwisi Helen Mavhandu-Mudzusi

**Affiliations:** Department of Health Studies, University of South Africa, Pretoria, South Africa

**Keywords:** advanced HIV disease, ART-experienced, ART-naïve, severe advanced HIV disease, very severe advanced HIV disease

## Abstract

**Background:**

Globally, about 630,000 (500,000–820,000) deaths occur annually among people living with HIV (PLHIV). Advanced HIV disease (AHD) continues to be an important contributor to HIV-related mortality in sub-Saharan Africa. Despite a significant positive association with mortality, the prevalence and factors that lead to AHD in Addis Ababa have not been studied well. Thus, this study aimed at investigating the prevalence and contributing factors of AHD in Addis Ababa, Ethiopia.

**Methods:**

We conducted a facility-based cross-sectional study among PLHIV attending Zewditu Memorial Hospital in Addis Ababa, Ethiopia. Data were collected through an interviewer-administered questionnaire and abstraction of 414 sturdy participants’ medical records using Open Data Kit. In the current study, AHD was defined using the objective criterion of a CD4 count < 200 cells/μL. Analysis was done using SPSS version 28 for descriptive and analytic statistics, and statistical significance was set at *p* < 0.05.

**Results:**

The overall prevalence of AHD, as well as among ART-naive and ART-experienced individuals, was 29.7% (95% CI: 25.4–34.4%), 51.4% (95% CI: 34.4–68.1%), and 27.6% (95% CI: 23.1–32.4%), respectively. Very severe and severe AHD accounts for 21.9% (95% CI: 15.0–30.3%) and 17.9% (95% CI: 11.5–25.8%) of the study participants, respectively. Further analysis of the determinants revealed that AHD was more likely to manifest in men (AOR of 2.41, 95% CI: 1.23–4.74); manual and service workers such as daily laborers, drivers, cleaners, and security guards (AOR of 2.75, 95% CI: 1.18–6.40); those with lower average monthly incomes of 5,001–10,000 birr (AOR of 3.27, 95% CI: 1.11–9.64) and less than 5,000 birr (AOR of 4.14, 95% CI: 1.49–11.54); the malnourished individuals (AOR of 5.82, 95% CI: 1.97–17.18); and those non-adherent to ART (AOR of 9.88, 95% CI: 4.32–22.03).

**Conclusion:**

The burden of AHD remains substantial, disproportionately affecting ART-naïve individuals. Male sex, low monthly income, being a manual and service worker, malnutrition, and ART non-adherence were key factors associated with HIV progression. Implementing the WHO-recommended AHD package may help reduce AHD and improve clinical outcome and ultimately death.

## Introduction

Human immunodeficiency virus (HIV) continues to claim the lives of 630,000 (ranging from 500,000 to 820,000) individuals per year. An average of one individual dies due to HIV-related causes per minute (1). In addition, 8.5% of people living with HIV (PLHIV) on ART experienced mortality and a CD4 count below 200 cells/μL, indicative of AHD, was a strong predictor of mortality (2). In 2024, it was estimated that Ethiopia would experience around 9,356 deaths yearly due to Acquired Immunodeficiency Syndrome (AIDS)-related causes, of whom 8,385 (89.6%) are adults aged 15 years and older (3). The same report indicated that Addis Ababa accounted for almost one-fifth (19.2%) of fatalities among PLHIV.

This high mortality is due to causes associated with advanced HIV disease (AHD), as there is evidence indicating that the majority of PLHIV die due to AHD (3, 4). In addition, there is evidence that demonstrates the mortality risk for PLHIV with AHD is nearly three times more than for those without it, with an adjusted odds ratio (AOR) of 2.77 (95% CI: 1.30, 5.92) (5). Nearly one in every three PLHIV had AHD at the time of diagnosis (6). Globally, there were an estimated 4.3 million (IQR 3.0–4.8) PLHIV who had AHD, defined as a CD4 count of 200 cells/μL or less. Of them, the majority, or 2.5 million, were from sub-Saharan African (SSA) countries, representing 58% of the global burden of AHD (7). A study in four high HIV burden SSA countries (Cameroon, Mozambique, Uganda, and Zimbabwe) revealed that an average of 16.1% of PLHIV had AHD (8). Moreover, the proportion of PLHIV with AHD in South Africa who were ART-naive and ART-experienced was estimated to be 58.6% (95% CI: 55.7–61.5%) and 43.5% (95% CI: 40.1–46.8%), respectively (9).

The World Health Organization (WHO) highlights the importance of early identification and management; however, many PLHIV still present with AHD, particularly in resource-limited settings. Despite a significant positive association between mortality and AHD, there is limited evidence on the extent of AHD and factors that contribute to its progression to it in Addis Ababa. In addition, evidence suggests that PLHIV with severe AHD, especially those with very severe AHD (CD4 less than 50 cells/μL), need considerable care, since their mortality rate is threefold greater than that of PLHIV without it (10). However, evidence regarding the severity of AHD remains limited, constraining a more comprehensive understanding of its clinical presentation, progression, and implications for patient management in Addis Ababa, Ethiopia. This research gap limits comprehension of the actual burden and contributing factors of AHD across various groups, therefore impeding the formulation of a policy for prevention, early diagnosis and management of AHD. Thus, this study focused on investigating the burden of AHD, categorized by ART experience, as well as the extent of AHD severity and its explanatory factors in Addis Ababa, with the goal of formulating measures to prevent the progression of HIV infection to its advanced stage.

## Materials and methods

### Study setting

The current study was carried out in Addis Ababa, the capital and largest city of Ethiopia. HIV predictions indicated that 18.3% (111,270) of Ethiopia’s 609,349 PLHIV in 2022 live in Addis Ababa (11). In 2024, a total of 103,319 PLHIV received ART across all health facilities in Addis Ababa (3). AHD services were primarily provided in public hospitals within the city where the necessary diagnostic capacity was available. The six hospitals from which our sample was drawn provide ART services and are affiliated with the Addis Ababa City Administration Health Bureau (AACAHB).

### Study design and population

The current study employed a cross-sectional study design among PLHIV aged 18 years or older, regardless of their ART status, who have a documented CD4 count visiting the health facility from May to October 2024.

### Sample size and sampling procedure

The sample size of 421 was determined based on existing literature, considering an AHD prevalence of 46.8% and associated factors of 65.5% (12, 13), with an anticipated 10% non-response rate. Zewditu Memorial Hospital was selected randomly. The hospital serves over 7,600 PLHIV, predominantly adults (98% of PLHIV on ART), who constituted the study population. The study subjects were selected using a systematic random selection method. Historical data showed that 1,550 PLHIV had a CD4 count over 6 months. The sampling interval (*k* = 4) was calculated by dividing the sampling frame (*N*′ = 1,550) by the sample size (421). To begin data collection, the first study participant selected randomly from 1 to 4 (k). Then, every fourth study participant was approached for data collection during the clinical visit. If the selected study participant did not meet the eligibility criteria, the next eligible participant was contacted.

### Definition of key terms

#### Advanced HIV disease

In this study, a CD4 count of <200 cells/μL was used as the sole criterion to objectively classify the presence or absence of AHD. WHO also recommends using clinical stages III and IV to define AHD when CD4 testing is unavailable (6).

#### Severe and very severe AHD

In adults severe AHD is defined as having a CD4 cell count between 50 and 100 CD4 cells/μL. Very severe AHD is characterized by a CD4 cell count < 50 cells/μL (6).

#### Adherence

The present study primarily evaluated adherence to treatment by comparing the prescribed ART dose and schedule with self-reported adherence. Good adherence: ≥95% of the dose, fair adherence: 85–94% of the dose, and poor adherence: <85% of the dose (14).

#### Nutritional status

It reflects the body’s nutritional condition and is assessed using body mass index (BMI). Adults are classified as malnourished if BMI < 18.5 kg/m^2^, normal if 18.5–24.9 kg/m^2^, and overweight/obese if ≥25 kg/m^2^ (14).

#### Occupation

Occupational categories with low frequencies (<5%) were merged during analysis based on the International Labour Organization; accordingly, daily laborers, drivers, cleaners, security guards, and similar occupations were grouped as manual and service workers (15).

#### Study variables

The dependent variable was AHD, and the independent variables were sociodemographic, economic, clinical, and treatment-related factors.

#### Data collection methods and procedures

Following obtaining consent, the study respondents were interviewed by using an interviewer-administered questionnaire to collect demographic and socioeconomic characteristics, including occupation, income, and education level. After the interview, the data encoders retrieved the study participants’ medical records using the unique ART (UART) number and medical record number (MRN) and extracted all pertinent clinical information using Open Data Kit (ODK). To protect confidentiality, no identifying information, including names, was collected from study participants.

#### Data quality assurance

A questionnaire from previous studies was customized for the current context and translated into the local language (Amharic). Training material was developed, and a two-day training was provided for data collectors. Supervision persisted throughout the data collection period. The data’s completeness was checked, and computerized data cleaning was conducted.

#### Data processing and analysis

The data from ODK was exported into SPSS version 28.0. Point and interval estimates were analyzed for frequency distribution, as well as bivariate and multivariate logistic regression. All statistical significance tests used a cut-off *p*-value < 0.05. Collinearity among significant variables was evaluated using *p*-values and correlation (r) before multivariable analysis.

#### Ethics statement

This study adhered to ethical principles to ensure the protection of respondents. Initially, voluntary involvement was strictly mandated, informed consent was assured, and written consent was obtained from each study participant. Ethical approval for the study was obtained from the UNISA College of Human Sciences Research Ethics Review Committee on April 2, 2024, under reference number 13709224CREC2024. The researchers received approval from the Ethical Clearance Committee of the City Government of Addis Ababa Health Bureau, reference number 15486/227. The study prioritized participant well-being by ensuring respondents had acess to clinical support services, particularly those with severe and very severe AHD.

## Results

### Basic sociodemographic and socioeconomic factors

A total of 414 study respondents with a response rate of 98.3% were enrolled in the study. Three of them refused to grant consent, two stated they do not have time to attend the interview, and two discontinued before completing key sections. The mean age and standard deviation (SD) are 44.31 (±12.17) years. As presented in Table 1, the study participants were predominantly female (58.5%), aged between 45 and 54 (35.3%), single (37.8%), had completed secondary education (40.9%), were self-employed (31.0%) and earned an average monthly income of ≤5,000 ($88) (34.2%).

**Table 1:** Basic sociodemographic and socioeconomic characteristics of the study participants, Addis Ababa, Ethiopia.

Variables (*n* = 414)		Frequency	Percentage (%)
Age (*n*’ = 414)			
	18–24	37	8.9
	25–34	61	14.7
	35–44	98	23.7
	45–54	146	35.3
	55+	72	17.4
Sex (*n*’ = 414)			
	Female	242	58.5
	Male	172	41.5
Marital status (*n*’ = 405)			
	Single	153	37.8
	Married	143	35.3
	Divorced/separated	69	17.0
	Widowed	40	9.9
Level of education (*n*’ = 401)			
	No Education	35	8.7
	Primary	145	36.2
	Secondary	164	40.9
	Tertiary	57	14.2
Occupation (*n*’ = 378)			
	Self-employment/merchant	117	31.0
	Employee (governmental/NGO/private)	89	23.5
	Students	22	5.8
	Housewives	32	8.5
	Jobless	63	16.7
	Others*	55	14.6
Average monthly income in birr (*n*’ = 383)			
	No income	57	14.9
	≤5,000 (≤$88)	131	34.2
	5,001–10,000 ($89–175)	121	31.6
	10,001 + ($ ≥ 176)	74	19.3

The sample size (*n*’) varies due to missing values. Others*: mainly daily laborers/drivers/cleaners/security guards.

### Prevalence of AHD

**Figure 1:**
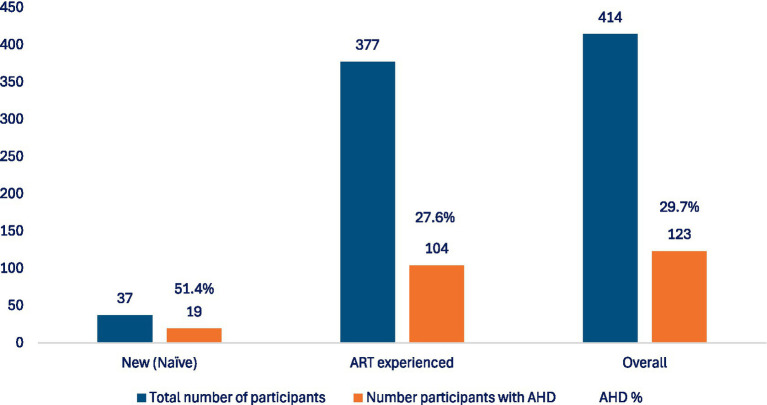
Overall AHD prevalence and AHD among ART-naive vs. ART-experienced study participants, Addis Ababa, Ethiopia.

**Figure 2:**
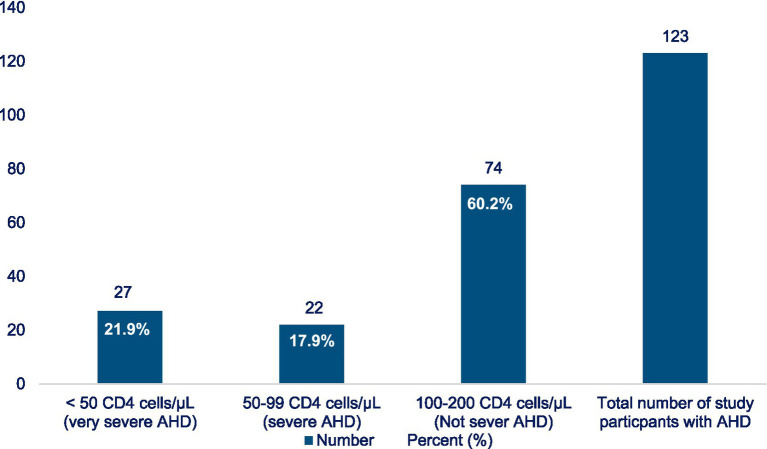
Prevalence of very severe (<50 CD4 cells/μL of blood) and severe (50–99 CD4 cells/μL) AHD among the study participants, Addis Ababa, Ethiopia.

As demonstrated in Figure 1, the overall prevalence of AHD was 29.7% (95% CI: 25.4, 34.4%). More than a quarter of the study respondents, specifically 27.6% (95% CI: 23.1–32.4%), who were ART-experienced, and 51.4% (95% CI: 34.4–68.1%), who were ART-naive, had AHD. One-fifth, 21.9% (95% CI: 15.0–30.3%), exhibited very severe AHD, followed by 17.9% (95% CI: 11.5–25.8%) with severe AHD (see Figure 2 for details).

### Clinical characteristics

A significant majority, 91.7%, were classified as WHO stage I and II; two-thirds, or 66.1%, demonstrated a normal nutritional status. As demonstrated in Table 2, the study participants were predominantly on ART (81.2%), had good adherence to ART (83.5%), had a history of opportunistic infections (54.3%), had been on ART for 16 years or more (33.8%) and were on the first-line regimen (92.5%).

**Table 2:** Clinical and treatment characteristics of the study participants, Addis Ababa, Ethiopia.

Variables (*n* = 414)		Frequency	Percentage (%)
WHO staging (current) (*n*’ = 407)			
	Stage I and II	377	91.7
	Stage III and IV	34	8.3
Nutritional status (current) (*n*’ = 389)			
	Normal (BMI: 18.5–24.9 kg/m^2^)	257	66.1
	Malnutrition (BMI < 18.5 kg/m^2^)	33	8.5
	Overweight/obese (BMI ≥ 25 kg/m^2^)	99	25.4
Follow-up status (*n*’ = 414)			
	On ART	336	81.2
	Transfer in	29	7.0
	Restart	12	2.9
	ART naïve	37	8.9
Adherence to treatment (*n*’ = 407)			
	Good (>95%)	340	83.5
	Fair (85–94%)	39	16.5
	Poor (<85%)	28	6.9
Duration on treatment in years (*n*’ = 414)			
	≤1 year	60	14.5
	2–5 years	58	14.0
	6–10 years	70	16.9
	10–15 years	86	20.8
	16+ years	140	33.8
Regimen (*n*’ = 412)			
	First-line	381	92.5
	Second line	26	6.3
	Third line	5	1.2
Past opportunistic infection			
	No	189	45.7
	Yes	225	54.3

The sample size (*n*’) varies due to missing values. Others*: Herpes zoster, skin rash or toxoplasmosis.

### Bivariate analysis

The independent variables, including sex, occupation, average monthly income, functional status, nutritional status, treatment adherence, follow-up status, and treatment duration, demonstrated a statistically significant association in the crude analysis with AHD at a *p*-value < 0.05, as presented in Table 3.

**Table 3:** Bivariate analysis of explanatory factors with advanced HIV disease among study participants, Addis Ababa, Ethiopia, expressed as crude odds ratios (COR) with 95% confidence intervals (95% CI).

Variables		Advanced HIV disease (AHD)		Crude OR (95% CI)	*p*-value
		Yes Number (%)	No Number (%)		
Sex					
	Female	59 (24.4%)	183 (75.6%)	1.00	
	Male	64 (37.2%)	108 (62.8%)	1.84 (1.20, 2.82)*	0.005*
Age					
	18–24	10 (27.0%)	27 (73.0%)	1.00	
	25–34	19 (31.1%)	42 (68.9%)	1.22 (0.49, 3.02)	0.665
	35–44	35 (35.7%)	63 (64.3%)	1.50 (0.65, 3.46)	0.341
	45–54	37 (25.3%)	109 (74.7%)	0.92 (0.41, 2.07)	0.834
	55+	22 (30.6%)	50 (69.4%)	1.19 (0.49, 2.87)	0.702
Marital status					
	Single	50 (32.7%)	103 (67.3%)	1.498 (0.90, 2.49)	0.120
	Married	35 (24.5%)	108 (75.5%)	1.00	
	Divorced/separated	24 (34.8%)	45 (65.2%)	1.646 (0.88, 3.08)	0.118
	Widowed	12 (30.0%)	28 (70.0%)	1.322 (0.61, 2.87)	0.480
Level of education					
	No education	13 (37.1%)	22 (62.9%)	1.29 (0.53, 3.11)	0.584
	Primary	44 (30.3%)	101 (69.7%)	0.94 (0.49, 1.83)	0.864
	Secondary	45 (27.4%)	119 (72.6%)	0.82 (0.49, 1.58)	0.551
	Tertiary	18 (31.6%)	39 (68.4%)	1.00	
Occupation					
	Self-employed	36 (30.8%)	81 (69.2%)	1.00	
	Employee	12 (13.5%)	77 (86.5%)	0.35 (0.17, 0.72)*	0.005*
	Student	6 (27.3%)	16 (72.7%)	0.84 (0.30, 2.33)	0.743
	Housewives	9 (28.1%)	23 (71.9%)	0.88 (0.37, 2.09)	0.773
	Jobless	22 (34.9%)	41 (65.1%)	1.21 (0.63, 2.31)	0.570
	Others**	29 (52.7%)	26 (47.3%)	2.51 (1.29, 4.85)*	0.006*
Average monthly income					
	≤5,000 birr ($88)	68 (36.2%)	120 (63.8%)	3.25 (1.60, 6.58)*	0.001*
	5,001–10,000 birr ($89–175)	36 (29.8%)	85 (70.2%)	2.43 (1.15, 5.13)*	0.021*
	10,001+ (≥$176)	11 (14.9%)	63 (85.1%)	1.00	
Past opportunistic infection					
	No	133 (70.4%)	56 (29.6%)	1.00	
	Yes	158 (70.2%)	67 (29.8%)	1.01 (0.66, 1.54)	0.974
Functional status (baseline)					
	Ambulatory	18 (30.5%)	41 (69.5%)	0.88 (0.48, 1.63)	0.690
	Bedridden	18 (19.4%)	75 (80.6%)	0.48 (0.27, 0.89)*	0.013*
	Working	87 (33.2%)	175 (66.8%)	1.00	
Nutritional status (current)					
	Normal	78 (30.4%)	179 (69.6%)	1.00	
	Malnutrition	21 (63.6%)	12 (36.4%)	4.02 (1.88, 8.57)*	<0.001*
	Overweight/obese	16 (16.2%)	83 (83.8%)	0.44 (0.24, 0.81)*	0.007*
Adherence to ART					
	Good (≥95%)	75 (22.1%)	265 (77.9%)	1.00	
	Fair/ poor (≤94%)	42 (62.7%)	25 (37.3%)	5.94 (3.34, 10.37)*	<0.001*
Follow-up status					
	Transferred in	15 (51.7%)	14 (48.3%)	1.00	
	On ART	81 (24.1%)	255 (75.9%)	0.29 (0.14, 0.64)*	0.002*
	Restart ART	8 (66.7%)	4 (33.3%)	1.87 (0.46, 7.60)	0.384
	ART-naive	19 (51.4%)	18 (48.6%)	0.99 (0.37, 2.61)	0.976
Duration of treatment in years					
	≤1 year	24 (40.0%)	36 (60.0%)	2.08 (1.10, 3.96)	0.026*
	2–5 years	21 (36.2%)	37 (63.8%)	1.77 (0.91, 3.43)	0.090
	6–10 years	24 (34.3%)	46 (65.7%)	1.63 (0.87, 3.04)	0.128
	10–15 years	20 (23.3%)	66 (76.7%)	0.95 (0.50, 1.78)	0.860
	16+ years	34 (24.3%)	106 (75.7%)	1.00	
Regimen					
	First line	114 (29.9%)	267 (70.1%)	1.00	
	Second/third line	8 (25.8%)	23 (74.2%)	0.82 (0.35, 1.88)	0.630

Others*: mainly daily laborers/ drivers/ cleaners/ security guards.

1:00: Reference group. *Statistically significant.

### Multivariate analysis

No collinearity was observed among the independent variables. After adjusting for the possible confounding variables in the logistic regression model, AHD was more likely to manifest in men (AOR of 2.41, 95% CI: 1.23–4.74), manual and service workers (AOR of 2.75, 95% CI: 1.18–6.40), lower average monthly incomes of 5,001–10,000 birr (AOR of 3.27, 95% CI: 1.11–9.64) and less than 5,000 birr (AOR of 4.14, 95% CI: 1.49–11.54), malnourished (AOR of 5.82, 95% CI: 1.97–17.18) and ART non-adherent (AOR of 9.88, 95% CI: 4.32–22.03) (see Table 4 for details).

**Table 4:** Multivariate analysis of explanatory factors with AHD among the study participants, Addis Ababa, Ethiopia, expressed as adjusted odds ratios (AOR) with 95% confidence intervals (95% CI).

Variables		Advanced HIV disease (AHD)		Crude	Adjusted	*p*-value
		Yes Number (%)	No Number (%)	OR (95% CI)	OR (95% CI)	
Sex						
	Female	59 (24.4%)	183 (75.6%)	1.00	1.00	
	Male	64 (37.2%)	108 (62.8%)	1.84 (1.20, 2.82)*	2.41 (1.23, 4.74)*	0.014*
Occupation						
	Own business	36 (30.8%)	81 (69.2%)	1.00	1.00	
	Employee	12 (13.5%)	77 (86.5%)	0.35 (0.17, 0.72)*	0.26 (0.09, 0.71)*	0.008*
	Student	6 (27.3%)	16 (72.7%)	0.84 (0.30, 2.33)	0.57 (0.15, 2.22)	0.421
	Housewives	9 (28.1%)	23 (71.9%)	0.88 (0.37, 2.09)	1.53 (0.47, 4.93)	0.478
	Jobless	22 (34.9%)	41 (65.1%)	1.21 (0.63, 2.31)	0.65 (0.25, 1.69)	0.378
	Others**	29 (52.7%)	26 (47.3%)	2.51 (1.29, 4.85)*	2.75 (1.18, 6.40)*	0.019*
Average monthly income						
	≤5,000 (≤$88)	68 (36.2%)	120 (63.8%)	3.25 (1.60, 6.58)*	4.14 (1.49, 11.54)*	0.007*
	5,001–10,000 ($89–175)	36 (29.8%)	85 (70.2%)	2.43 (1.15, 5.13)*	3.27 (1.11, 9.64)*	0.032*
	10,001+ ($ ≥ 176)	11 (14.9%)	63 (85.1%)	1.00	1.00	
Functional status (intake/baseline)						
	Ambulatory	18 (30.5%)	41 (69.5%)	0.88 (0.48, 1.63)	1.12 (0.47, 2.68)	0.803
	Bedridden	18 (19.4%)	75 (80.6%)	0.48 (0.27, 0.89)*	0.47 (0.20, 1.12)	0.089
	Working	87 (33.2%)	175 (66.8%)	1.00	1.00	
Nutritional status (current)						
	Normal	78 (30.4%)	179 (69.6%)	1.00	1.00	
	Malnutrition	21 (63.6%)	12 (36.4%)	4.02 (1.88, 8.57)*	5.82 (1.97, 17.18)	0.001*
	Overweight	16 (16.2%)	83 (83.8%)	0.44 (0.24, 0.81)*	0.79 (0.33, 1.85)	0.181
Adherence to treatment						
	Good (≥95%)	75 (22.1%)	265 (77.9%)	1.00	1.00	
	Fair/poor (≤94%)	42 (62.7%)	25 (37.3%)	5.94 (3.34, 10.37)*	9.88 (4.32, 22.03)*	<0.001*
Follow-up status						
	Transferred in	15 (51.7%)	14 (48.3%)	1.00	1.00	
	On ART	81 (24.1%)	255 (75.9%)	0.29 (0.14, 0.64)*	0.63 (0.19, 2.09)	0.453
	Restart	8 (66.7%)	4 (33.3%)	1.87 (0.46, 7.60)	3.33 (0.42, 26.47)	0.255
	New	19 (51.4%)	18 (48.6%)	0.99 (0.37, 2.61)	5.54 (0.57, 53.42)	0.139
Duration on treatment						
	≤1 year	24 (40.0%)	36 (60.0%)	2.08 (1.10, 3.96)*	0.37 (0.06, 2.17)	0.270
	2–5 years	21 (36.2%)	37 (63.8%)	1.77 (0.91, 3.43)	1.38 (0.56, 3.39)	0.481
	6–10 years	24 (34.3%)	46 (65.7%)	1.63 (0.87, 3.04)	1.09 (0.46, 2.56)	0.836
	10–15 years	20 (23.3%)	66 (76.7%)	0.95 (0.50, 1.78)	0.98 (0.40, 2.44)	0.972
	16+ years	34 (24.3%)	106 (75.7%)	1.00	1.00	

Others*: mainly daily laborers/drivers/cleaners/security guards.

1:00: Reference group. *Statistically significant association with dependent variable.

## Discussion

The prevalence of AHD was 29.7% (95% CI: 25.4–34.4%), higher among ART-naïve PLHIV (51.4, 95% CI: 34.4–68.1%). AHD in this study was defined using CD4 alone, whereas WHO definition also includes clinical stage III or IV (6). As combined definitions capture additional cases, our approach may have led to a lower estimate of AHD prevalence. More than one-fifth had very severe AHD (21.9, 95% CI: 15.0–30.3%), while 17.9% (95% CI: 11.5–25.8%) had severe AHD. Male sex (AOR: 1.84, 95% CI: 1.20–2.82), lower income (5,001–10,000 birr; AOR: 4.28, 95% CI: 1.24–14.78 and <5,000 birr; AOR: 6.84, 95% CI: 2.06–22.66), engagement in manual or service occupations (AOR: 4.42, 95% CI: 1.68–11.60), malnutrition (AOR: 5.82, 95% CI: 1.97–17.18), and fair or poor ART adherence (AOR: 9.88, 95% CI: 4.32–22.03) were all independently associated with increased odds of AHD.

The study demonstrated a high overall prevalence of AHD. The current finding is consistent with evidence from WHO and other global reports, indicating that nearly one-third of PLHIV present with AHD, with a disproportionately higher burden in SSA and other low- and middle-income settings (6). It exceeds findings in Cameroon, Mozambique, Zimbabwe, and Uganda, which recorded 28.8, 16.6, 19.2, and 14.0%, respectively (8). It was substantially higher than the aggregate AHD (9.7–17%) in Kenya, Malawi, South Africa, and Zambia (16, 17). It was significantly lower than the results of Adama, Ethiopia (63.8%) (18). The variation is partly due to a difference in ART status, as studies suggest that up to 42% more ART-naïve PLHIV may develop AHD compared to those who have ART experience (9, 18). The alternative explanation for the variations may be the WHO staging versus CD4 testing for AHD classification (19) in which WHO staging is less accurate than CD4 for AHD diagnosis (20).

AHD was observed in more than half of ART-naïve PLHIV. The interval estimates of this study are comparable to the 43.5% prevalence of AHD among ART-naïve PLHIV in South Africa (9). It was much lower than Tanzania, Kenya, and Adama (Ethiopia), where 62–80% of ART naïve PLHIV developed AHD (18, 19, 21). This disparity may be due to Tanzania and Kenya employing WHO staging or CD4 count, whereas Adama is a seriously sick PLHIV. However, this study included all adult PLHIV with CD4 counts, regardless of illness severity. The current finding was higher than Uganda, Kenya, South Africa, and Ghana, which found 35.1, 35.4, 33, and 28.6%, respectively (10, 12, 22, 23). Of the ART-experienced study participants, 27.6% (95% CI: 23.1–32.4%) had AHD. There is limited evidence on AHD among ART-experienced PLHIV. The present result was lower than Cameroon’s (39.8%) and Malawi, Kenya, and South Africa’s (30%) (16, 24). It surpassed the other Kenyan study, which indicated that 20% of ART-experienced PLHIV had AHD (19). Treatment interruption duration may explain the disparities, as longer ART interruptions increase the probability of immunological failure (25), which leads to AHD.

The study found that a notable proportion of participants had severe and very severe AHD. Few studies have examined severe and very severe AHD. The present study’s finding was comparable to recent aggregate data from four African nations, including Ethiopia, showing 16.7% of participants had severe AHD (26). It showed less severe AHD than three African countries, where 27.0% had it (16). It surpassed a four-country study involving Ethiopia, Kenya, Mozambique, and Tanzania that found 14.4% of PLHIV had severe AHD (26). Very severe AHD was lower than in a study in Tanzania, which found 27.1% (21). The variation may be due to the study subgroup, since ART-naïve people exhibited high rates of severe and very severe AHD. Previous studies in Malawi, South Africa, and Kenya found that 65% of PLHIV were ART-naïve (16). In contrast, only 8.9% of the individuals in the current study were ART naïve.

As we examine the contribution of sociodemographic factors to AHD, male gender had a significant association with AHD. The current finding is comparable with prior studies conducted in Kenya, South Africa, Nigeria and Tanzania (12, 13, 21, 25, 26). Moreover, evidence from Southern Ethiopia reveals significant disparities, with males demonstrating higher levels of AHD (66% of males compared to 56% of females, *p*-value < 0.001) (27). This imbalance may be attributable to men often delaying treatment until major symptoms manifest. In Malawi, a qualitative study found that HIV-positive men were less likely to initiate ART than women (28).

The findings indicate that lower income levels were progressively accompanying with greater HIV progression to AHD, reflecting a negative dose–response association. There is insufficient data on the influence of income on the progression of HIV infection to AHD. The present finding was confirmed by another study, which revealed that those in the lowest income quartiles exhibited a 1.9 times higher probability of having AHD (29). The current finding is comparable to a previous study that the likelihood of AHD increases with lower average income (30, 31). On the contrary, the previous studies found that financial resources did not affect HIV infection progression (22, 32). This discrepancy might be due to financial factors that can have varying influences or consequences depending on the specific circumstances and context.

Manual and service workers (such as daily laborers) had a greater probability of AHD than the self-employed. This result is consistent with prior findings showing PLHIV in similar occupational categories had a higher likelihood of having AHD (30, 31, 33). Compared to self-employed study participants, employees had a reduced likelihood of AHD at AOR 0.17 (95% CI: 0.06–0.53) and a *p*-value of 0.002. The previous study also shows that employed individuals are less likely to have AHD (33). A steadier lifestyle may help employed people to take better care of themselves and their drugs than business owners.

Malnourished individuals had a greater probability of AHD than those with normal nutritional status. In addition to the present finding, earlier studies in low-income and SSA countries, including Nigeria and Tanzania, found that malnutrition accelerates HIV infection progression (21, 28, 34). A study conducted in Southern Ethiopia also found that PLHIV with low BMIs, indicating malnutrition, had lower mean CD4 cell counts, suggestive of AHD (35). The explanation could be that nutrition boosts immunological resistance and overall health (36, 37). On the other hand, malnutrition reduces immune resilience, undermining overall health and the ability to control HIV infection progression to AHD.

Despite exhibiting a broad CI, potentially attributed to the insufficient sample size for this variable, study participants with fair and poor adherence to ART demonstrated a significant likelihood of having AHD in comparison to those who had a good adherence to treatment. Various previous studies conducted in different African countries support the current findings (20, 23, 24). Furthermore, the present finding was consistent with another study conducted in Southern Ethiopia (35). Negative self-image, a lack of belief in the need for ART, alcohol usage, treatment exhaustion, and stigmatization, among other factors, may all lead to drug non-adherence in Africa and similar contexts (23, 38), such as Addis Ababa.

## Strengths and limitations

The strength of the current study, unlike prior studies, is categorizing AHD severity prevalence into two distinct groups: very severe (CD4 count < 50 cells/μL) and severe (CD4 count 50–100 cells/μL). This will help prioritize severe AHD interventions, allocate resources, and make programming decisions. A key limitation of the study is the wide CI for AHD among ART-naïve PLHIV, along with nutritional status and adherence, which may be attributed to low sample size. Given the cross-sectional design, causal inferences between AHD and factors such as income, occupation, nutritional status, and adherence cannot be made.

## Conclusion

The study found a significant prevalence of AHD among ART-naïve and ART-experienced PLHIV in Addis Ababa, Ethiopia. Nearly 30% of PLHIV had AHD, and severe AHD affected nearly two-fifths of the study respondents, with a disproportionate impact on ART-naïve individuals. However, it is also significant among those experienced with ART and calls for urgent intervention. The current study identified significant sociodemographic, economic, and clinical factors that influence the progression of HIV. AHD was independently associated with male gender, low monthly income, and manual and service workers such as daily laborers. Malnourished and non-ART-adherent individuals exhibited an elevated likelihood of AHD.

Despite expanded access to antiretroviral therapy, AHD remains a significant challenge. Strengthening the implementation of the WHO-recommended AHD care package, including rapid ART initiation and continuity, key diagnostics (CD4, tuberculosis, and cryptococcal antigen tests), and preventive therapies, is essential to reduce morbidity and mortality. The study findings will contribute to the body of knowledge related to advanced HIV disease in public health. The study’s findings may assist in the formulation of policy objectives and enable context-specific targeted strategies to improve AHD prevention and management practices in Ethiopia and similar contexts. HIV programming should prioritize a differentiated service delivery model for males and particular occupational categories for the prevention of AHD. In addition, the study recommended the Ministry of Health and stakeholders strengthen a multisectoral approach to empower PLHIV to address low economic status, low-level occupational status, treatment adherence, and malnutrition.

## Data Availability

The original contributions presented in the study are included in the article/supplementary material, further inquiries can be directed to the corresponding author.
